# Persistently high plasma procalcitonin levels despite successful treatment of tuberculous pleuritis and tuberculous lymphadenitis patients

**DOI:** 10.1038/s41598-024-71627-5

**Published:** 2024-09-29

**Authors:** Zaib un Nisa, Atiqa Ambreen, Tehmina Mustafa

**Affiliations:** 1 Department of Pathology, Gulab Devi Hospital, Lahore, Pakistan; 2 Department of Microbiology, Faculty of Science & Technology, University of Central Punjab, Lahore, Pakistan; 3 Department of Microbiology, Gulab Devi Hospital, Lahore, Pakistan; 4 Center for International Health, Department of Global Public Health and Primary Care, University of Bergen, Bergen, Norway; 5 Department of Thoracic Medicine, Haukeland University Hospital, Bergen, Norway

**Keywords:** Procalcitonin, TB lymphadenitis, TB pleuritis, Response to treatment, Inflammatory biomarkers, Biomarkers, Diseases, Medical research

## Abstract

In a prospective cohort study, we evaluated plasma PCT levels in 48 TB lymphadenitis (TBLN) and 41 TB pleuritis (TBPE) patients. Measurements of PCT were done in unstimulated plasma of microbiologically and clinically confirmed TBLN and TBPE patients registered for anti-TB treatment at a tertiary care hospital in Lahore, Pakistan. Plasma levels of PCT were found to be raised in 89% of the patients at baseline with a median of 1.5 ng/ml. Levels were higher (*p* = 0.001) in TBLN as compared to TBPE (2.69, 0.96 ng/ml). PCT levels were not related to the bacterial burden depicted by culture positivity in these patients. PCT showed a negative correlation with the severity of constitutional symptoms (rho = − 0.238, *p* = 0.034), and inflammatory biomarkers; ferritin (rho = − 0.43,* p* < 0.001), INF-γ (rho = − 0.314,* p* = 0.003), TNF-α (rho = − 0.220,* p* = 0.039), IL-6 (rho = − 0.224,* p* = 0.035), and several chemokines of CCL and CCXL group. Raised plasma levels of PCT did not decrease with anti-TB treatment, indicating it is not a good biomarker to monitor treatment response in TBLN and TBPE patients. More studies with a larger number of confirmed EPTB cases are needed to define the role of PCT and its interaction with other biomarkers in EPTB.

## Introduction

Procalcitonin (PCT) is a 116-amino acid molecule and precursor of calcitonin produced by thyroid gland^1^. Its diagnostic potential for identifying systemic bacterial infections became evident in 1993 when it was observed that PCT levels are elevated in patients with bacterial infections and sepsis^2^. The extra-thyroid synthesis of PCT occurs in various tissues but is suppressed in the absence of bacterial infection^3^. However, in the presence of bacterial endotoxins or inflammatory cytokines such as IL-6, TNF-α, and IL-1β, PCT synthesis increases substantially, up to 100 to 1000 times higher, indicating its role as a biomarker for inflammation and bacterial infections^4^. Furthermore, the reduction in PCT levels following appropriate antibiotic treatment highlighted its value as a biomarker for monitoring treatment response in bacterial infections. Use of PCT in clinical decisions has reduced the overuse of antibiotics^5^. In contrast, the down-regulation of PCT production is documented by cytokines such as IFN-ꝩ. Infection with *Mycobacterium tuberculosis* (Mtb) induces production of IFN-ꝩ along with other inflammatory cytokines which is thought to be reason for low levels of PCT in tuberculosis (TB) as compared to other bacterial infections^6,7^. Some studies on pulmonary TB have reported PCT levels < 0.5 ng/ml^8–10^. There is a paucity of literature describing levels of PCT in extrapulmonary TB (EPTB). There are a few case reports showing normal PCT levels in cutaneous TB (PCT = 0.055 ng/ml) or slightly raised levels in TB pleuritis (PCT = 0.27 ng/ml) and TB pericarditis (PCT < 0.5 ng/ml)^11–13^. Little is known about the dynamics of PCT secretion in EPTB in response to anti-TB treatment. This study aimed to evaluate the role of PCT in TB lymphadenitis (TBLN) and TB pleuritis (TBPE) patients and its utility to be used as a biomarker to monitor treatment response in these two common manifestations of EPTB. Moreover, it aimed to explore the relationship between PCT and other inflammatory biomarkers produced during TB disease.

## Results

### Patient characteristics

**Fig. 1:**
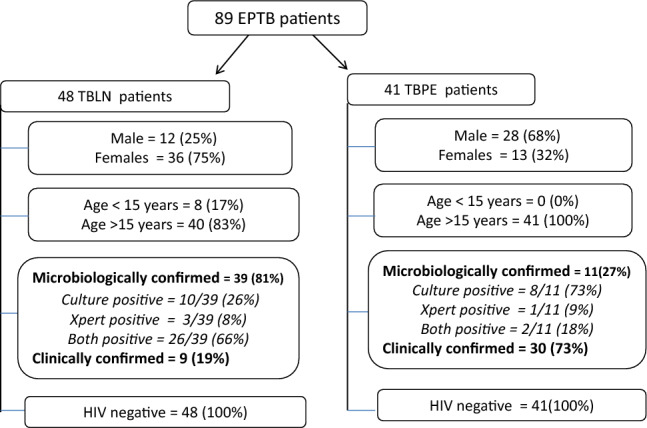
Flow chart showing patients included in the study and their demographic and clinical characteristics. EPTB: extra-pulmonary tuberculosis, TBLN: tuberculous lymphadenitis, TBPE: tuberculous pleuritis, HIV: human immunodeficiency virus.

Figure 1 shows the demographic characteristics of the enrolled patients. A total of 89 EPTB patients were included in the study and were followed up at 2 and 6 months of anti-TB treatment. Out of these, 48/89 (54%) had TBLN, and 41/89 (46%) had TBPE. A significantly higher proportion of females 36/48 (75%) was observed among TBLN patients, while males 28/41 (68%) predominated among TBPE cases (*p* < 0.001). All TBPE patients were adults (> 15 years), whereas, eight TBLN patients were < 15 years of age. A total of 50/89 (56%) were microbiologically confirmed TB cases by culture and/or Xpert, while 39/89 (44%) were clinically confirmed TB cases based on the composite reference standard. There were significantly more culture-confirmed cases among TBLN (39/48) as compared to TBPE (11/41) patients (*p* < 0.001). All patients tested negative for HIV.

### Procalcitonin levels before treatment

**Fig. 2:**
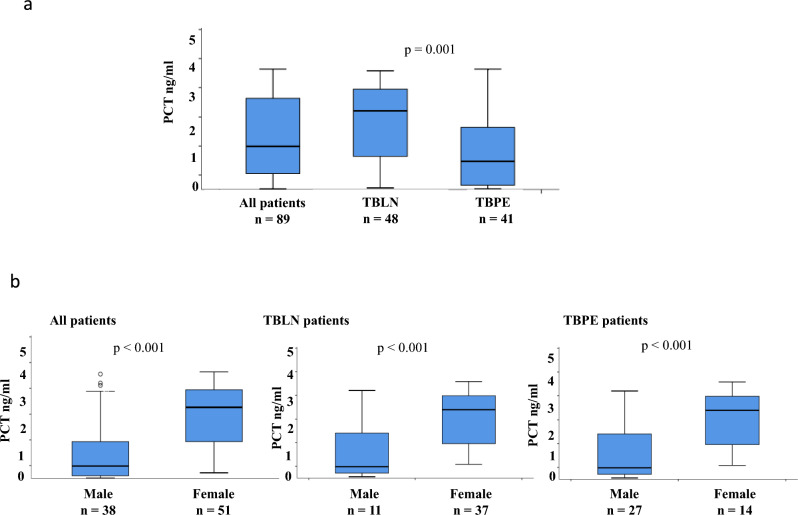
Box plots showing plasma levels of plasma procalcitonin in, a) all patients, tuberculous lymphadenitis, and pleuritis patients, and b) male and female patients. The Mann–Whitney U was used to compare procalcitonin levels among tuberculous lymphadenitis, and pleuritis patients, and male and female patients in each group. A *p-*value < 0.05 was considered significant. Boxes represent the median and interquartile range, and the whisker shows minimum/maximum values. PCT: procalcitonin, TBLN: tuberculous lymphadenitis, TBPE: tuberculous pleuritis, n = number of patients in each group.

Figure 2a shows that the median level of PCT among all patients was 1.5 ng/ml (range: 0.02–4.14 ng/ml) at baseline. The levels were significantly higher (*p* = 0.001) in TBLN patients with a median of 2.69 ng/ml (range: 0.05–4.07 ng/ml) as compared to the TBPE patients (median of 0.96 ng/ml; range: 0.02–4.14 ng/ml). When compared among male and female patients separately (Fig. 2b), the levels of PCT were significantly higher (*p* < 0.001) in females with a median of 2.6 ng/ml (range: 0.02–4.14 ng/ml) as compared to male patients (median: 1.1 ng/ml, range: 0.03–4.0 ng/ml). Similar findings were observed when analyzed separately in TBLN and TBPE (Fig. 2b).

Five patients reported co-morbid conditions (hypertension, chronic liver disease and diabetes). PCT levels were not significantly different between patients with and without co-morbid conditions.

**Fig. 3:**
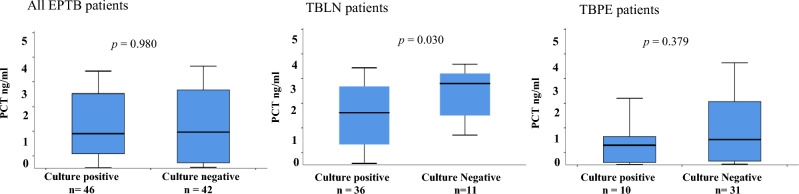
Box plots showing difference of plasma procalcitonin levels in culture-positive and culture-negative cases among all patients, TB lymphadenitis, and TB pleuritis patients. The Mann–Whitney U was used to compare procalcitonin levels among the culture-positive and culture-negative patients in each group. A *p-*value < 0.05 was considered significant. Boxes represent the median and interquartile range, and the whisker shows minimum/maximum values. PCT: procalcitonin, EPTB: extra-pulmonary tuberculosis, TBLN: tuberculous lymphadenitis, TBPE: tuberculous pleuritis, n = number of patients at different time points.

Figure 3 shows the levels of PCT between culture-positive and culture-negative patients. No significant difference (*p* = 0.709) was observed in PCT levels among all patients. When analyzed separately culture-negative TBLN patients had significantly raised PCT levels (median of 3.3 ng/ml; range: 1.2–4.1 ng/ml) as compared to the culture-positive (median of 2.1 ng/ml; range: 0.05–3.9 ng/ml) cases (*p* = 0.028). Among TBPE cases PCT levels were not significantly different among culture-positive (median of 0.79 ng/ml; range: 0.02–2.7 ng/ml) and culture-negative (median of 1.03 ng/ml; range: 0.03–4.1 ng/ml) patients (*p* = 0.379).

### Association of PCT with symptom burden at baseline

**Fig. 4:**
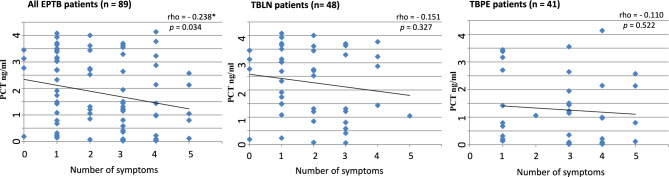
Dot plots showing association of symptom burden with plasma concentration of PCT in all, TBLN, and TBPE patients. Each dot represents a patient’s plasma PCT concentration and the number of symptoms reported by the patients. The Spearman correlation test was used to see the association among PCT levels and number of symptoms. *Correlation is significant at the 0.05 level (2-tailed). A *p-*value < 0.05 was considered significant. PCT: procalcitonin, EPTB: extra-pulmonary tuberculosis, TBLN: tuberculous lymphadenitis, TBPE: tuberculous pleuritis, n = number of patients.

Figure 4 shows correlation of PCT levels at baseline with the number of constitutional symptoms reported by the patients. Among all patients a weak negative correlation was seen between PCT levels and the number of constitutional symptoms (rho = − 0.238, *p* = 0.034). When analyzed separately levels of PCT in TBLN (rho = − 0.151, *p* = 0.327) and TBPE (rho = − 0.110, *p* = 0.522) showed a non-significant negative correlation trend with the number of constitutional symptoms.

**Fig. 5:**
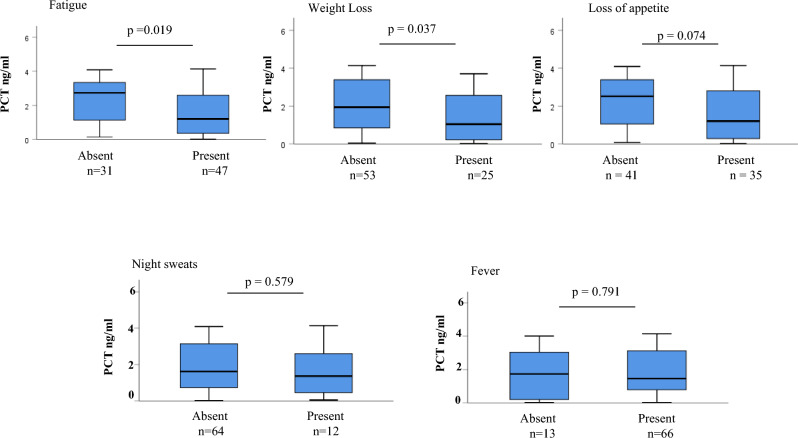
Box plots showing difference in plasma levels of PCT in patients with different constitutional symptoms. The Mann–Whitney U was used to compare PCT levels in patients with or without the presence of constitutional symptoms. A *p-*value < 0.05 was considered significant. Boxes represent the median and interquartile range, and the whisker shows minimum/maximum values. PCT: procalcitonin, n = number of patients at different time points.

Figure 5 shows the association of presence or absence of individual symptoms with plasma PCT levels. The plasma PCT levels were significantly less in patients showing weight loss (*p* = 0.037) and fatigue (*p* = 0.019) as compared to the patients with no history of weight loss and fatigue. A non-significant negative trend was also seen in patients with loss of appetite (*p* = 0.074). No significant difference in PCT levels was seen among patients having night sweats (*p* = 0.579) and fever (*p* = 0.791) as compared to the patients without these two symptoms.

### Correlation of PCT levels with ferritin, CRP, ADA and a panel of 40-plex Human Chemokine Panel of inflammatory biomarkers

Table 1 shows the correlation of PCT levels with ferritin, ADA, CRP, and a panel of 40 different inflammatory immune biomarkers studied earlier on the same patients before treatment and at 6 months of treatment. Among all patients, plasma PCT levels showed a significant negative correlation with ferritin (rho = − 0.43,* p* < 0.001), INF-γ (rho = − 0.314,* p* = 0.003), TNF-α (rho = − 0.220,* p* = 0.039), GM-CSF (rho = − 0.241,* p* = 0.025), IL1-β (rho = − 0.378,* p* < 0.001), IL2 (rho = − 0.228,* p* = 0.035), IL-6 (rho = − 0.224,* p* = 0.035), IL-10 (rho = − 0.324,* p* = 0.002), and multiple chemokines of CCL (CCL1,CCL2, CCL8, CCL13, CCL17, CCL19, CCL20, CCL25, CCL26, CCL27), and CXCL group (CXCL2, CXCL6, CXCL11). At 6 months of treatment PCT levels showed a significant negative correlation with only one chemokine (CCL26), while: non-significant negative correlation trend was seen with most of the studied inflammatory biomarkers.

**Table 1:** Correlations of plasma PCT levels with ferritin, CRP, ADA, and a panel of 40-plex Human Chemokine Panel of inflammatory biomarkers.

Biomarkers	Procalcitonin					
	Before treatment			After 6 months of treatment		
	All patients N = 89	TBLN N = 48	TBPE N = 41	All patients N = 73	TBLN N = 40	TBPE N = 33
Ferritin	− 0.439**	− 0.370*	− 0.212	− 0.195	− 0.115	− 0.164
CRP	− 0.184	− 0.01	0.063	0.124	0.298	0.152
ADA	0.104	0.323*	− 0.138	− 0.057	− 0.165	0.092
INF-ꝩ	− 0.312**	− 0.224	− 0.342*	− 0.038	− 0.148	0.041
TNF-α	− 0.225*	− 0.249	− 0.104	− 0.033	− 0.135	0.113
GM-CSF	− 0.242*	− 0.193	− 0.155	− 0.087	− 0.189	0.111
IL-1β	− 0.364**	− 0.317*	− 0.421**	0.034	− 0.043	0.236
IL 2	− 0.223*	− 0.121	− 0.256	− 0.083	− 0.221	0.128
IL6	− 0.233*	− 0.15	− 0.216	0.104	− 0.086	0.314
IL 10	− 0.316**	− 0.27	− .357*	− 0.197	− 0.294	− 0.145
CCL1	− 0.274*	− 0.215	− 0.147	− 0.065	− 0.157	0.025
CCL 2	− 0.219*	− 0.282	− 0.116	− 0.061	− 0.186	0.198
CCL 8	− 0.349**	− .412**	− 0.091	0.03	− 0.096	0.146
CCL 13	− 0.216*	− 0.28	− 0.13	0.004	− 0.068	0.151
CCL 17	− 0.228*	− 0.242	− 0.142	0.073	− 0.03	0.179
CCL 19	− 0.388**	− .375**	− .406**	− 0.197	− 0.192	− 0.261
CCL 20	− 0.247*	− 0.22	− 0.224	0.035	0.008	0.096
CCL 25	− 0.289**	− 0.185	− 0.314*	− 0.048	− 0.192	0.091
CCL 26	− 0.319**	− 0.310*	− 0.348*	− 0.354**	− 0.373*	− 0.406*
CCL 27	− 0.224*	− 0.186	− 0.093	0.093	0.25	0.055
CXCL 2	− 0.243*	− 0.184	− 0.144	0.132	0.075	0.208
CXCL6	− 0.377**	− .457**	− 0.186	− 0.004	− 0.145	0.106
CXCL 11	− 0.263*	− 0.139	− 0.144	0.039	0.054	0.089
MIF	− 0.007	0.117	− 0.043	0.121	− 0.013	0.468**
IL 4	− 0.138	− 0.178	− 0.03	− 0.022	− 0.13	0.202
IL 8	− 0.091	− 0.132	− 0.088	0.109	0.096	0.191
IL 16	− 0.069	0.006	− 0.207	0.078	− 0.102	0.380*
CCL 3	− 0.205	− 0.075	− 0.181	0.079	0.064	0.152
CCL 7	− 0.173	− 0.015	− 0.21	0.133	− 0.013	0.394*
CCL 11	− 0.177	− 0.112	− 0.151	− 0.063	− 0.167	0.113
CCL 15	− 0.142	− 0.12	− 0.096	− 0.06	− 0.041	0.036
CCL21	− 0.171	− 0.096	− 0.143	− 0.202	− 0.15	− 0.26
CCL 22	− 0.058	− 0.147	0.058	0.1	0.016	0.173
CCL 23	− 0.081	− 0.071	0.12	0.119	0.084	0.207
CCL 24	− 0.158	− 0.208	− 0.039	− 0.005	0.036	0.104
CXCL 1	− 0.098	− 0.137	0.045	0.067	− 0.003	0.15
CXCL 5	− 0.162	− 0.145	− 0.285	− 0.301*	− 0.339*	− 0.384*
CXCL9	− 0.202	− 0.174	− 0.044	0.237*	0.129	0.463**
CXCL10	− 0.193	− 0.136	− 0.022	0.142	0.104	0.264
CXCL12	− 0.166	− 0.123	− 0.088	0.115	− 0.047	0.283
CXCL 13	− 0.183	− 0.101	− 0.225	0.252*	0.362*	0.109
CXCL 16	− 0.08	− 0.235	0.061	0.022	− 0.125	0.248
CX3CL1	− 0.167	− 0.107	− 0.154	0.009	0.029	0.056

*Correlation is significant at the 0.05 level (2-tailed).

**Correlation is significant at the 0.01 level (2-tailed).

The Spearman correlation test was used to see the correlation between PCT and other inflammatory biomarkers.

### Effect of treatment on PCT levels

**Fig. 6:**
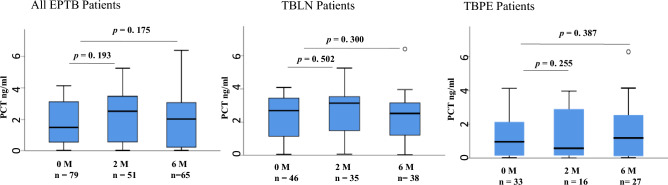
Box plots showing all extra-pulmonary, TB lymphadenitis, and TB pleuritis patients with raised plasma levels of procalcitonin at baseline and changes in its levels at 2 and 6 months of treatment. The Wilcoxon signed rank test was used to compare biomarkers expression at different time points. A *p-*value < 0.05 was considered significant. Boxes represent the median and interquartile range, and the whisker shows minimum/maximum values. PCT: procalcitonin, EPTB: extra-pulmonary tuberculosis, TBLN: tuberculous lymphadenitis, TBPE: tuberculous pleuritis, n = number of patients at different time points. M: month of treatment.

PCT levels were raised in 89% (79/89) of all patients. Figure 6 shows plasma PCT levels of patients with raised values before treatment did not decrease significantly at both 2 (*p* = 0.175) and 6 months (*p* = 0.193) of treatment. Among patients with TBLN, PCT levels were raised in 96% (46/48) of cases, no significant change was observed in plasma levels of PCT with treatment at both 2 (*p* = 0.502) and 6 months (*p* = 0.300) in these patients. Among TBPE cases 80% (33/41) had elevated PCT levels before the start of the treatment. Similarly no significant change was observed in plasma levels of PCT at both 2 (*p* = 0.255) and 6 months (*p* = 0.387) of treatment in these patients.

## Discussion

We describe the levels of PCT in 89 HIV negative EPTB (48 TBLN and 41 TBPE) patients. Plasma levels of PCT were raised in 89% of our patients with a median of 1.5 ng/ml. The median levels of PCT in TBLN (2.68 ng/ml) were significantly higher than TBPE (median, 0.96 ng/ml) patients. These findings suggest that different presentations of EPTB may have different host responses reflected in the differential expression of inflammatory markers. Our previous work on these patients also show that host responses of TBLN and TBPE are different reflected in the difference of biomarkers decreasing in response to treatment^14,15^. A recent review also concluded that different presentations of TB have different host immune responses and the cytokines and chemokines produced in response to Mtb and their interactions are important in the clinical presentation of the disease^16^.

To understand the interaction of PCT with different inflammatory biomarkers, PCT levels were correlated with plasma levels of CRP, ferritin, ADA, and 40 different inflammatory immune biomarkers studied earlier on the same patients^14,15^. Among all patients, plasma levels of PCT negatively correlated with most of inflammatory biomarkers notably ferritin, INF-γ, TNF-α, GMCSF, IL1-β, IL2, IL6, and IL10. IFN- ꝩ is known to down regulate PCT release and secretion of IFN-ꝩ has been thought of  as a reason why PCT levels are not raised in viral and fungal infections and low levels of PCT in these infections are used to exclude bacterial infections^17–20^.

There are no similar studies on humans evaluating correlation of PCT with inflammatory biomarkers such as IFN-ꝩ in TB. An animal study on dogs evaluating relationship of PCT with different inflammatory biomarkers (TNF-α, IL-1β, IL-6, and IFN-γ) produced during viral, bacterial, and parasitic infections showed that PCT showed a weak significant negative correlation with IFN-ꝩ levels in serum. IL-1β, and IL6 showed a non-significant negative trend, while a non-significant positive trend was seen with TNF-α^21^. Our findings are similar to this study and imply that IFN-ꝩ down regulates the production of PCT and this may be the cause of negative correlations of PCT with other inflammatory biomarkers.

When compared with constitutional symptoms, PCT levels correlated negatively with the number of constitutional symptoms in our patients, and this may be attributed to increased pro-inflammatory biomarkers in patients with more constitutional symptoms.

Although PCT levels are expected to be low in viral infections, raised levels were demonstrated in viral sepsis in the absence of bacterial infections and it was proposed as a marker of sepsis rather an indication of bacterial infections^22^. Alternatively low levels of PCT may be taken as an indication of mild disease, rather than ruling out bacterial infections^23^. The PCT levels are also reported to be increased in infections caused by gram negative bacteria as compared to gram positive bacteria^20,24^. However, it is shown that many aspects of PCT pathophysiology are still unknown. A retrospective of analysis of 168 hospitalized patients from USA admitted in different wards and intensive care units showed that PCT levels did not correlate with disease severity in bacterial infections and were not different among gram negative and gram positive infections. Similarly PCT levels did not correlate with mortality and other unfavorable outcomes of the studied population. It was however associated with high bacterial burden (positive blood cultures for gram-negative and gram-positive bacteria)^25^. We found no association with culture positivity among all EPTB and TBPE patients; however, high PCT levels were seen in culture-negative TBLN patients as compared to culture-positive TBLN cases. These findings do not support PCT as a simple biomarker indicating disease severity or bacterial burden. It has complex interactions with other inflammatory biomarkers and these interactions shape the final plasma levels of PCT and unless these interactions are clearly understood PCT levels cannot be used in EPTB to assess disease severity. This highlights the fact that the immune responses in different presentations of TB are different and one size fit all approach cannot be applied to the immune responses of all presentations of EPTB. More studies needed to explore the specific mechanisms or implications of PCT levels in the context of disease.

Plasma levels of PCT were raised in 89% of our patients; making it a good candidate biomarker for monitoring response to treatment. However, when patients were followed longitudinally and plasma samples tested for PCT at 2 and 6 months of anti-TB treatment PCT levels did not significantly decrease in our cohort of patients. Present literature on utility of PCT in monitoring response to treatment in TB has reported its limited value as a prognostic marker. A study from Japan assessed the ability of PCT levels to predict mortality risk in 252 pulmonary patients out of which 22 had associated EPTB (10 TBPE, 3 TBLN, 5 intestinal TB, and 4 trachea/bronchus TB) among survivors (n = 213) and non-survivors (n = 39). The levels of PCT were measured at day zero, 7, 14, and 28 of diagnosis of TB and initiation of treatment. Although levels of PCT were significantly raised in non-survivors at each time point, no significant difference in decline of PCT levels was reported among survivors or non-survivors with treatment^26^. Similarly a study from India recruiting 55 children with pulmonary TB demonstrated no change in PCT levels after second month of treatment^27^. Another recent case report shows sustained increased levels of PCT in a dialysis patient diagnosed with TBLN without sepsis. The levels remained high (2.9 ng/ml) after anti-TB treatment and clinical improvement in patient’s condition^28^. In our cohort the median PCT in TBLN at 6 months was 2.56 ng/ml. The elevated serum PCT levels, initially high at diagnosis and remaining so after the commencement of anti-TB treatment may indicate non-TB inflammatory processes affecting PCT release. This heightened inflammation might arise from various factors, including the eicosanoid network, lipid metabolism, autophagy, or other inflammatory mechanisms^29^. A study including 319 culture-confirmed pulmonary TB patients evaluated role of 70 biomarkers (including PCT) in TB disease severity and response to treatment. Disease severity was assessed by presence of any cavities, cavities > 4 cm, and > 50% involvement of lungs on X-rays, smear positivity grade, and turn-around time of culture positivity on MGIT. At base line PCT levels were significantly associated with only > 50% lung involvement on x-rays. Levels of PCT was seen to decrease at week 8, however, PCT levels did not show an association with microbiological treatment response (culture conversion at 8 and 12 weeks)^30^. The decrease in levels was calculated by changes in levels at week 8 compared to week zero and not decreases to normal values, alternatively, this may be the reflection of TB type and inclusion of only culture positive cases in the study. Leboueny et al. demonstrated a significant decrease in PCT in pulmonary TB patients after four weeks of anti-TB treatment. The study included only 21 pulmonary TB patients, with pre-treatment and post treatment PCT done for only nine patients as most of the patients did not return for follow-up. This contradictory finding could be due the small number of patients included in the analysis^31^. An interesting study by Chendi et al. compared levels of inflammatory biomarkers in TB endemic (South Africa) and non-endemic (Norway) areas and reported low levels of PCT in pulmonary TB as compared to other respiratory diseases in patients from low TB-endemic area, while, in patients from TB endemic area PCT levels was raised in TB patients as compared to other respiratory diseases. Norwegian patients were also followed longitudinally and a decrease in levels of PCT was seen after second month of treatment^8^. The South African cohort was not followed longitudinally to see changes in PCT levels with treatment. However, another earlier study from South Africa evaluating 74 host biomarkers on a multiplex platform for TB diagnosis and monitoring treatment response in pulmonary TB patients, and reported no change in PCT levels with treatment^32^. We also report raised levels of PCT not decreasing with treatment from a TB-endemic area. It has been suggested before that, levels of PCT are affected by geographic area and ethnicity. Host Inflammatory biomarkers against TB are shown to be associated with ethnic variation of the infected individuals^33,34^. In our study, females had higher levels of PCT as compared to males. Thus, role of PCT in TB seems to be affected by the site of disease, prevalence of disease in the population, gender and ethnicity of the patients.

Our study has some limitations; A total of 50/89 (56%) of our patients were microbiologically confirmed TB cases, culture quantification was not documented by the laboratory and results were reported as either positive or negative, this limitation could affect true correlations between the levels of PCT and the bacterial load. Rest of the patients 39/89 (44%) included in the study were not culture confirmed cases and this may have resulted in wrong categorization of patients as TB cases. However it is very difficult to bacteriologically confirm all EPTB cases due to paucibacillary nature of the disease even where culture facilities are available. In routine clinical scenarios, EPTB patients are clinically diagnosed. Moreover, all clinically diagnosed cases in our cohort had supporting laboratory diagnosis (histology consistent with TB for TBLN and lymphocytosis, protein levels exceeding 3 g/dl, or concurrent pulmonary TB indicated by positive acid-fast bacilli smear and/or chest radiograph for TBPE cases) and a good response to the anti-TB treatment as defined in the composite reference standard. These are good criteria to diagnose a TB case in the absence of microbiological confirmation. Secondly a considerable number of patients were excluded from the analysis due to non-availability of plasma samples at baseline or at 2 and/or 6 months of treatment as some patients did not turn-up for the follow-up visits or did not consent to give blood samples for research purpose. Another limitation is that long term follow-up was not done to predict role of PCT in relapse or return to normal levels. Further studies involving different presentations of confirmed TB cases from different epidemiological areas are needed to answer the controversies related to PCT in TB.

## Conclusions

PCT levels were slight/moderately raised in TBLN and TBPE patients and showed negative correlation with severity of symptoms and other pro-inflammatory biomarkers. Raised plasma levels of PCT did not decrease with successful anti-TB treatment, indicating it is not a good biomarker to monitor treatment response in TBLN and TBPE. More studies including different types of TB patients and long term follow-up are needed to confirm these results and define the role of PCT in TB.

## Methods

We conducted a prospective longitudinal cohort study from April 2016 to August 2017. The study site was Gulab Devi Hospital Lahore, Pakistan, a tertiary care facility diagnosing and treating TB patients. All presumptive EPTB cases of any age visiting outpatient department at the study site, who underwent the routine diagnostic work-up for TB and started on anti-TB treatment, were included in the study. All patients were followed up until completion of treatment. Written informed consent was obtained from all study participants before the start of the study. All experiments were performed in accordance with relevant guidelines and regulations.

### Sampling

Five ml of blood samples were collected at three distinct time points before start of treatment (0 months), 2 months, and 6 months post-treatment using EDTA vacutainer. Plasma was separated and transferred to − 80 °C until required for further analysis.

Patients with enlarged lymph nodes underwent excision biopsy and biopsy material was sent for histopathology and microbiological examination. Pleural fluids were aspirated from TBPE patients. The aspirated fluids were sent for cytology and microbiological evaluation.

### Sample processing

Lymph node biopsies and pleural fluids were subjected to routine TB diagnostic workup including smears for acid-fast bacilli detection with Ziehl–Neelsen (ZN) and auramine staining methods, the Xpert MTB/RIF assay (Xpert) for Mtb DNA detection, and solid and automated liquid media for mycobacterial cultures^35^. Auramine O-stained smears were examined using a fluorescence microscope with light emitting diode illumination^36^. The Xpert assay was performed following the manufacturer's instructions^37^. For culture, Lowenstein Jensen medium (two slopes) and a Mycobacteria Growth Indicator Tube (MGIT) 960TM from Becton Dickinson, Sparks, MD, USA, were used^35^.

### Procalcitonin levels measurement in plasma

The plasma levels of PCT were measured by an enzyme-linked immune-sorbent assay (ELISA), as per manufacturer’s instructions (Elabscience^®^) based on the principle of solid phase sandwich ELISA^38^. The cut-off value for PCT was taken as 0.1 ng/ml^39^.

### Ferritin, CRP, and ADA measurement in plasma

Plasma levels of ferritin and CRP were measured by the ELISA^40,41^ and ADA levels by the kinetic chemistry method^42^ according to the manufacturers’ protocol as described earlier^15^.

### Inflammatory biomarkers estimation in plasma

A 40-plex human cytokine/chemokine panel (Biorad 40 plex Bio-PlexPro^TM^Human Chemokine Panel) was used on a multiplex platform to detect inflammatory biomarkers in the plasma samples as described earlier^14^.

### Classification of patients/case definition

Patients were classified using a composite reference standard based on a combination of clinical, radiological, and laboratory findings, resulting in two groups: “microbiologically confirmed" and clinically confirmed TBLN and TBPE cases. A “microbiologically confirmed" TBLN or TBPE case was defined as one where a lymph node biopsy specimen or pleural fluid was positive on culture and/or Xpert. A “clinically confirmed TBPE" case included patients with symptoms and findings consistent with TB pleuritis, pleural fluid examination showing lymphocytosis and protein levels exceeding 3 g/dl, or concurrent pulmonary TB indicated by positive acid-fast bacilli smear and/or chest radiograph, and who showed a good response to the anti-TB treatment. A “clinically confirmed TBLN" case included patients with symptoms, clinical findings, and histopathology findings consistent with TBLN, and who showed a good response to the anti-TB treatment. A good response to anti-TB treatment was defined as the presence of at least two of the following three criteria, (i) improvement in the symptoms reported by the patients, (ii) improvement in local signs of the disease (e.g. regression of lymph nodes in lymphadenitis patients or reduction of pleural fluid volume on ultrasound in pleuritis cases), (iii) weight gain.

### Statistical analysis

Data analysis was done using IBM's Statistical Package for Social Sciences (SPSS) version 25. Statistical tests included the Chi-square test was to analyze categorical data, the Mann–Whitney U test for comparing two different groups, and Spearman rank test for correlation analysis, and the Wilcoxon signed rank test for comparing values at different time points. Statistical significance was established at p < 0.05.

## Data Availability

The case-based data are available from the corresponding author on reasonable request.
